# Science between Bioreactors and Space Research—Response to Comments by Joseph J. Bevelacqua et al. on “Dexamethasone Inhibits Spheroid Formation of Thyroid Cancer Cells Exposed to Simulated Microgravity”

**DOI:** 10.3390/cells9081763

**Published:** 2020-07-23

**Authors:** Marcus Krüger, Sascha Kopp, Markus Wehland, Thomas J. Corydon, Daniela Grimm

**Affiliations:** 1 Department of Microgravity and Translational Regenerative Medicine, Clinic for Plastic, Aesthetic and Hand Surgery, Otto von Guericke University, Universitätsplatz 2, 39106 Magdeburg, Germany; sascha.kopp@med.ovgu.de (S.K.); markus.wehland@med.ovgu.de (M.W.); dgg@biomed.au.dk (D.G.); 2 Research Group “Magdeburger Arbeitsgemeinschaft für Forschung unter Raumfahrt- und Schwerelosigkeitsbedingungen” (MARS), Otto von Guericke University, Universitätsplatz 2, 39106 Magdeburg, Germany; 3 Department of Biomedicine, Aarhus University, Høegh-Guldbergsgade 10, 8000 Aarhus C, Denmark; corydon@biomed.au.dk; 4 Department of Ophthalmology, Aarhus University Hospital, Palle Juul-Jensens Boulevard 167, 8200 Aarhus N, Denmark

We would like to thank Bevelacqua et al. for their interest in our article [1] and their comments [2]. We completely agree with the authors that the complex space environment cannot be adequately simulated on Earth. However, this was not our intention for these studies on tumor spheroids. The influence of space radiation would even be counterproductive in targeting a metastasis model to reflect the situation in cancer patients on Earth.

For many years, cells have been exposed to conditions of microgravity and scientists have investigated how these cells might sense or adapt to microgravity [3,4]. Growing three-dimensional cell aggregates in space and in laboratories using microgravity simulators is a typical application of microgravity research [5]. The NASA-developed rotating wall vessel bioreactor was specially applied to engineer such tissue constructs. The random positioning machine (RPM) works as a three-dimensional clinostat negating the directional influence of the *g*-vector providing simulated conditions of micro- or partial gravity (also referred as “time-averaged microgravity” by some authors). It should be noted and we are aware that simulated microgravity differs from real (space) microgravity in several aspects. Nevertheless, the RPM has become a well-established device for gravitational research [6,7], which is also recommended by international space agencies (like NASA [8] or the European Space Agency, ESA [9]) for experiments in reduced gravity. Although it is known to produce additional shear stress and fluid dynamics within the cell culture flasks [10,11], Wuest et al. also evaluated the RPM as a “reliable tool supporting ground-based microgravity studies” and an “ideal tool for preliminary microgravity tests, screening studies in which simulated microgravity effects are checked on various organisms” [12]. This is caused by the fact that cellular effects that were observed in real microgravity could be reproduced with good agreement on RPMs [13,14,15,16,17,18]. In this study, however, these questions were not of importance, as the RPM merely served as a tool for the generation of tumor spheroids. We believe that the RPM is superior to other methods of spheroid generation such as liquid overlay techniques or spinner flasks, as in this system it is possible to induce cells to detach from an already established cellular network and to form spheroids suspended in the culture medium. These spheroids, which were also found after long-term cultures in space [19], are an important model system to mimic micrometastases or microregions of solid tumors [20]. Despite their simplicity, tumor spheroids are often used for drug testing in pharmacology today [21,22,23] and are still state of the art. More complex tumor organoids co-cultured with different cell types are in development and will replace the homogenous spheroids in the future, better mimicking tumor biology in vivo. In addition, the investigation of microgravity-induced cell detachment and spheroid formation delivers valuable information about processes like metastasis and in vivo cancer progression [22]. The RPM-based metastasis model can be effectively used to study in vitro whether drugs can favor or inhibit spheroid formation. Of course, mechanical forces have to be taken into consideration while designing experiments with the RPM and analyzing their results. However, this can be done by using appropriate controls. Moreover, cells inside the human body, especially those in the process of forming metastases, are subjected to a multitude of forces besides gravity; therefore, the implementation of a completely shear-force-free experimental environment would not improve the results.

In summary, the arguments of Bevelacqua et al. [2] may result from misunderstandings of the actual purpose of the experiments. Not only has it never been the intention of this study to simulate space conditions in complete detail, it was never intended to simulate space at all and we never claimed to do so. Our aim was to investigate molecular mechanisms of metastasis development and the identification of possible target molecules for potential antimetastatic pharmacological interventions using a device that, by simulating certain aspects of microgravity, induces the formation of spheroids from originally adherently growing tumor cells, something that cannot be achieved as easily by other established methods. Some years ago Becker and Souza wrote: “Combination of the resources available in the unique environment of microgravity with the tools and advanced technologies that exist in laboratories across Earth may inform new research approaches to expand the knowledge necessary for improving treatment options, and enhancing the quality of life for those affected by this illness.” [24]. In our study, we used space-derived investigations to fight cancer on Earth.

We thank the editors for giving us the opportunity to provide a reply to the letter.

## References

[1] Melnik D., Sahana J., Corydon T.J., Kopp S., Nassef M.Z., Wehland M., Infanger M., Grimm D., Krüger M. (2020). Dexamethasone Inhibits Spheroid Formation of Thyroid Cancer Cells Exposed to Simulated Microgravity. Cells.

[2] Bevelacqua J., Welsh J., Mortazavi S. (2020). Comment on “Dexamethasone Inhibits Spheroid Formation of Thyroid Cancer Cells Exposed to Simulated Microgravity”. Cells.

[3] Ingber D. (1999). How cells (might) sense microgravity. FASEB J..

[4] Cogoli A., Tschopp A., Fuchs-Bislin P. (1984). Cell sensitivity to gravity. Science.

[5] Unsworth B.R., Lelkes P.I. (1998). Growing tissues in microgravity. Nat. Med..

[6] Herranz R., Anken R., Boonstra J., Braun M., Christianen P.C., de Geest M., Hauslage J., Hilbig R., Hill R.J., Lebert M. (2013). Ground-based facilities for simulation of microgravity: Organism-specific recommendations for their use, and recommended terminology. Astrobiology.

[7] Borst A.G., van Loon J.J.W.A. (2008). Technology and Developments for the Random Positioning Machine, RPM. Microgravity Sci. Technol..

[8] NASA Microgravity Simulation Support Facility. https://www.nasa.gov/sites/default/files/atoms/files/microgravity_simulation_support_facility_mssf_one_pager.pdf.

[9] ESA Ground-Based Facilities Programme. https://esamultimedia.esa.int/docs/HRE/ESA-CORA-GBF_final_rev.6.pdf.

[10] Wuest S.L., Stern P., Casartelli E., Egli M. (2017). Fluid Dynamics Appearing during Simulated Microgravity Using Random Positioning Machines. PLoS ONE.

[11] Hauslage J., Cevik V., Hemmersbach R. (2017). Pyrocystis noctiluca represents an excellent bioassay for shear forces induced in ground-based microgravity simulators (clinostat and random positioning machine). NPJ Microgravity.

[12] Wuest S.L., Richard S., Kopp S., Grimm D., Egli M. (2015). Simulated Microgravity: Critical Review on the Use of Random Positioning Machines for Mammalian Cell Culture. Biomed. Res. Int..

[13] Benavides Damm T., Walther I., Wüest S.L., Sekler J., Egli M. (2014). Cell cultivation under different gravitational loads using a novel random positioning incubator. Biotechnol. Bioeng..

[14] Wuest S.L., Richard S., Walther I., Furrer R., Anderegg R., Sekler J., Egli M. (2014). A Novel Microgravity Simulator Applicable for Three-Dimensional Cell Culturing. Microgravity Sci. Technol..

[15] Cogoli-Greuter M. (2014). The Lymphocyte Story—An Overview of Selected Highlights on the in Vitro Activation of Human Lymphocytes in Space. Microgravity Sci. Technol..

[16] Schwarzenberg M., Pippia P., Meloni M.A., Cossu G., Cogoli-Greuter M., Cogoli A. (1999). Signal transduction in T lymphocytes—A comparison of the data from space, the free fall machine and the random positioning machine. Adv. Space Res..

[17] Walther I., Pippia P., Meloni M.A., Turrini F., Mannu F., Cogoli A. (1998). Simulated microgravity inhibits the genetic expression of interleukin-2 and its receptor in mitogen-activated T lymphocytes. FEBS Lett..

[18] Villa A., Versari S., Maier J.A., Bradamante S. (2005). Cell behavior in simulated microgravity: A comparison of results obtained with RWV and RPM. Gravit. Space Biol. Bull..

[19] Pietsch J., Ma X., Wehland M., Aleshcheva G., Schwarzwälder A., Segerer J., Birlem M., Horn A., Bauer J., Infanger M. (2013). Spheroid formation of human thyroid cancer cells in an automated culturing system during the Shenzhou-8 Space mission. Biomaterials.

[20] Kunz-Schughart L.A. (1999). Multicellular tumor spheroids: Intermediates between monolayer culture and in vivo tumor. Cell Biol. Int..

[21] Krüger M., Melnik D., Kopp S., Buken C., Sahana J., Bauer J., Wehland M., Hemmersbach R., Corydon T.J., Infanger M. (2019). Fighting Thyroid Cancer with Microgravity Research. Int. J. Mol. Sci..

[22] Nunes A.S., Barros A.S., Costa E.C., Moreira A.F., Correia I.J. (2019). 3D tumor spheroids as in vitro models to mimic in vivo human solid tumors resistance to therapeutic drugs. Biotechnol. Bioeng..

[23] Sant S., Johnston P.A. (2017). The production of 3D tumor spheroids for cancer drug discovery. Drug Discov. Today Technol..

[24] Becker J.L., Souza G.R. (2013). Using space-based investigations to inform cancer research on Earth. Nat. Rev. Cancer.

